# The effect of state self-control on the intertemporal decisions made by individuals with high and low trait self-control

**DOI:** 10.1371/journal.pone.0195333

**Published:** 2018-04-03

**Authors:** Yuan Guan, Jiamei He

**Affiliations:** 1 Department of Psychology, Liaoning Normal University, Dalian, China; 2 Collaborative Innovation Center of Healthy Personality Assessment and Cultivation of Children and Adolescents in Liaoning Province, Dalian, China; Technion Israel Institute of Technology, ISRAEL

## Abstract

The present study aimed to explore how state self-control influences the intertemporal decisions made by individuals with high and low trait self-control. State self-control, represented by the degree of depletion, was manipulated by conducting Stroop tasks with different levels of difficulty, and the intertemporal decision task was used as a self-control task. Compared with participants with high trait self-control, the preferences of participants with low trait self-control for immediate rewards were more vulnerable to the difficulty of depletion tasks. Throughout the experimental stages, the heart rate variability (HRV) of participants with high trait self-control was significantly higher than that of participants with low trait self-control, indicating that individuals with high trait self-control may have stronger and more stable self-control abilities.

## Introduction

Self-control helps individuals adjust their mental and behavioral activities to achieve their goals [1–4]. Self-control plays an important role in intertemporal decisions [5–7] when the decision and its consequences occur at different times [8–10]. Whether individuals choose immediate rewards in smaller amounts or restrain their impulsivity to obtain future valuable rewards depends heavily on the process of self-control in intertemporal decisions [11–16]. Researchers have found that an individual’s failure of self-control leads to a higher preference for immediate rewards [5, 11–12, 17].

Research distinguishes between state self-control and dispositional self-control [18]. Substantial individual differences exist in people’s capacity for self-control, which is assumed to be relatively stable across situations and over time [19]. Individuals who are usually better able to manage their lives, hold their tempers and fulfill their promises are thought to be with high dispositional or trait self-control [18]. Compared with individuals with low trait self-control, those with high trait self-control are more patient and more likely to choose delayed rewards when making intertemporal decisions [13]. Heart rate variability (HRV) refers to the beat-to-beat variation in heart rate and reflects the continuous interplay between the sympathetic and parasympathetic systems in determining the heart rate [20–21]. The neurovisceral integration model [22–24] suggests that trait HRV (resting HRV) represents the inhibitory capacity of the central autonomic network (CAN) [25]. The CAN plays an important role in regulating behaviors, cognition and emotions and includes the prefrontal cortex (PFC), which is related to inhibitory and executive function, and the anterior cingulate cortex (ACC), which is involved in monitoring conflicts [20]. Studies on intertemporal decisions have shown that the cingulate gyrus and prefrontal cortex play an important self-control role in controlling and regulating individuals’ impulses for immediate gratification [26–28]. The cingulate cortex is responsible for monitoring the consistency between the subjective value of rewards and individuals’ goals, plans and needs [29] and for calculating the expected value of control and the cost of self-control [30]. The prefrontal cortex exerts top-down control [28, 30] to eliminate the deviation between subjective values and goals. Restricted activity in the prefrontal cortex may lead to failure of self-control when making intertemporal decisions [11, 27]. According to these studies, resting HRV may be related to the self-control capacity and could predict individuals’ performance in intertemporal decision tasks. Individuals with high trait self-control should exhibit a higher resting HRV and be better at inhibiting the impulse for immediate rewards when making intertemporal decisions than those with low trait self-control.

Individuals’ state self-control varies across situations and time [31]. State self-control is susceptible to situational influences, such as previous attempts at self-control [32–33]. It has been found that intertemporal decisions are affected by the state of self-control [34]. After accomplishing depletion tasks, people are temporarily in a lower self-control state [35–36]. For example, in some studies, participants have been asked to complete depletion tasks to manipulate the state of self-control, which is represented by the variation of the amount of self-regulatory resources available for intertemporal choice tasks. With the reduction in self-regulatory resources, it was more difficult for participants to resist the temptation of immediate rewards, and the proportion of participants who chose immediate rewards increased [34–35, 37]. In further research, the preferences for immediate rewards in intertemporal decisions were observed to be reduced among participants with supplemental self-regulatory resources [38]. Therefore, individuals’ intertemporal decisions are affected by their state self-control, and variations in self-regulatory resources caused by depletion tasks lead to variations in state self-control.

Overall, the inhibition of immediate gratification is related to participants’ trait self-control and state self-control. Compared with individuals with low trait self-control, individuals with high trait self-control were more patient and more likely to choose delayed rewards when making intertemporal decisions [13]. Individuals were more willing to choose immediate rewards after completing high-depletion tasks (which resulted in a lower self-control state) than after low-depletion tasks (which resulted in a higher self-control state) [34]. Although these previous studies have respectively discussed the effects of trait self-control [13] and state self-control [34] on intertemporal decisions, there has been no further discussion of how the two factors work together in intertemporal decision making. Therefore, whether the state of self-control has similar impacts on individuals with high trait self-control and individuals with low trait self-control is the main subject that will be discussed in the present work.

Previous studies have shown that individuals with low trait self-control are more prone to seek out information and situations that easily trigger their impulsivity than individuals with high trait self-control [14, 39]. Taking attention away from impulsive cues requires self-regulatory resources [40]. Therefore, when making intertemporal decisions, individuals with low trait self-control may need to consume more self-regulatory resources to suppress their attention to the immediate rewards than individuals with high trait self-control. This results in individuals with low trait self-control being more vulnerable to depletion tasks that cost self-regulatory resources and reduce their state of self-control.

Two hypotheses have been proposed in light of the above. Individuals with high trait self-control exhibit a higher resting HRV than individuals with low trait self-control. The preference for immediate rewards in individuals with low trait self-control is more vulnerable than in individuals with high trait self-control when the difficulty of depletion tasks is manipulated. The dual-task paradigm [35] was adopted in the present study, and the degree of depletion representing the state of self-control was manipulated by Stroop tasks. A physiological polygraph was used to record resting HRV and HRV in tasks during the experimental stages.

## Materials and methods

### Participants

The Chinese version of the self-control scale for college students was used (original version developed by Tangney [18] and revised by Tan [41]) as a screening tool to select participants with high and low trait self-control. This 5-point scale comprises 19 items. The items are reverse scored, in addition to the first, fifth, 11th and 14th items. High scores indicate a high level of trait self-control. In the present study, the Cronbach α coefficient of the scale was 0.85.

A total of 420 questionnaires were sent out, and 347 were received in return, with an effective recovery of 82.62%. To collect participants with significantly different levels of trait self-control, we selected participants who scored in the highest 27% (more than 72 points) and the lowest 27% (less than 58 points) in the list of scores into the high trait self-control group and the low trait self-control group, respectively [42]. An independent sample *t* test showed that the selected participants with high (*M* = 76.39, *SD* = 3.70) and low trait self-control (*M* = 52.53, *SD* = 3.77) had significantly different scores in the self-control scale (*t*_(160)_ = 40.62, *p* < 0.001, *d* = 6.39), indicating that they were effectively differentiated in trait self-control in our study. Twenty-three participants were unable to participate in the experiment for personal reasons, with 164 remaining participating participants. Half of the participants with high trait self-control and half with low trait self-control were randomly assigned to complete low-depletion tasks, and the other half were assigned to complete high-depletion tasks. Two participants were excluded from the statistical analysis because they did not understand the experimental process, according to our retrospective questions about the experimental process and their low reaction accuracy (less than 70%) in the Stroop task. Thus, a total of 162 participants (34 males and 128 females, aged 17 to 27 years, mean age = 20.78, *SD* = 2.42) were included in the study (Table 1); all were undergraduate or postgraduate students. Their vision or corrected vision was normal, with no color blindness or color weakness. All participants received payment and gave verbal informed consent. Two participants were under the age of 18 (2 females), and we obtained consent from their guardians by phone. All experimental procedures were approved by the Human Subjects Review Board of Liaoning Normal University’s School of Psychology in China.

**Table 1:** Number of males and females and age characteristics of the four groups.

			Males (*n*)	Females (*n*)	Age (*M ± SD*)
**High trait self-control**	**High depletion**	44	11	33	20.73 ± 2.39
	**Low depletion**	39	11	28	20.79 ± 2.73
**Low trait self-control**	**High depletion**	40	8	32	20.78 ± 2.33
	**Low depletion**	39	4	35	20.82 ± 2.30

### Experimental tasks

#### Depletion task

The Stroop task was adopted to manipulate the degree of depletion. Participants were asked to differentiate the painting color of words while suppressing the dominant response to process the meaning of the words, during which mental resources for self-control would be consumed [34, 37, 43]. According to Yuan [44] and the results of the preliminary test, we used the Chinese words for "red", "green", "yellow", "blue" and "HHH" as experimental materials, written in red, green, yellow or blue text. The high-depletion Stroop task included three types of stimuli: "word-color"-mismatched stimuli (e.g., the word "red" written in green text); "word-color"-matched stimuli (e.g., "red" written in red text) and neutral stimuli ("HHH" written in red, green, yellow, or blue text); the proportion of mismatched, matched, and neutral stimuli was 3:2:1, and there were 144 stimuli in total. The low-depletion Stroop task included two types of stimuli: 72 "word-color"-matched stimuli and 72 neutral stimuli.

**Fig 1:**
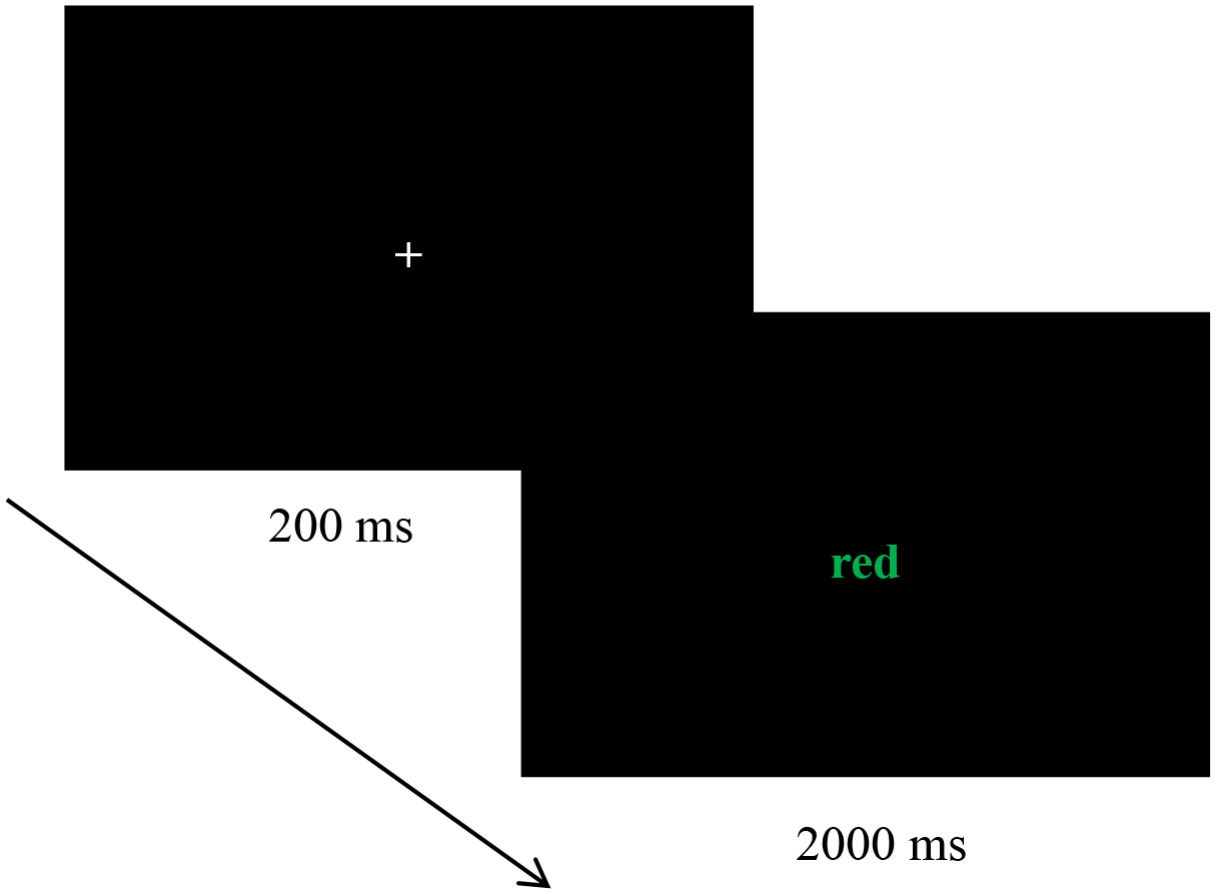
Process diagram of the Stroop task.

The Stroop tasks were programmed with E-prime 1.1 software. In each trial, the fixation was shown at the center of screen for 200 ms, and the target stimulus was then presented for 2000 ms. Participants were asked to react to the color of the target stimulus by pressing a button (the matches between the A, S, W and D buttons and the colors red, yellow, blue and green were balanced among participants) (Fig 1). If participants did not respond within 2000 ms, the experiment automatically advanced to the next trial. Target stimuli were presented pseudorandomly to prevent participants from exhibiting the same responses in three consecutive trials. Feedback was provided to participants only at the practice stage.

#### Intertemporal decision tasks

Participants were asked to make choices between smaller-but-sooner rewards and larger-but-longer rewards in intertemporal decision tasks. Immediate rewards included 18, 19, 20 or 21 Chinese Yuan available today. The amount of the delayed rewards was increased by 10~250% over the amount of the immediate rewards, and the time required for the delayed rewards was 7, 15 or 30 days [45] (Table 2). Each immediate reward was paired with a delay time of 7 days, 15 days and 30 days. There were ten delayed rewards for each pair of immediate and delayed time. Therefore, each immediate reward was paired with 30 delayed rewards, which were presented randomly. After 30 trials were presented, the next immediate reward was presented with 30 delayed rewards. Therefore, a total of 120 trials were performed.

**Table 2:** Percentage of increasing in delayed rewards compared with immediate rewards.

Days	Percentage increase (%) (delayed reward/immediate reward–1)									
7	10	15	20	30	50	70	90	125	140	170
15	15	20	30	50	70	90	125	140	170	200
30	20	30	50	70	90	125	140	170	200	250

**Fig 2:**
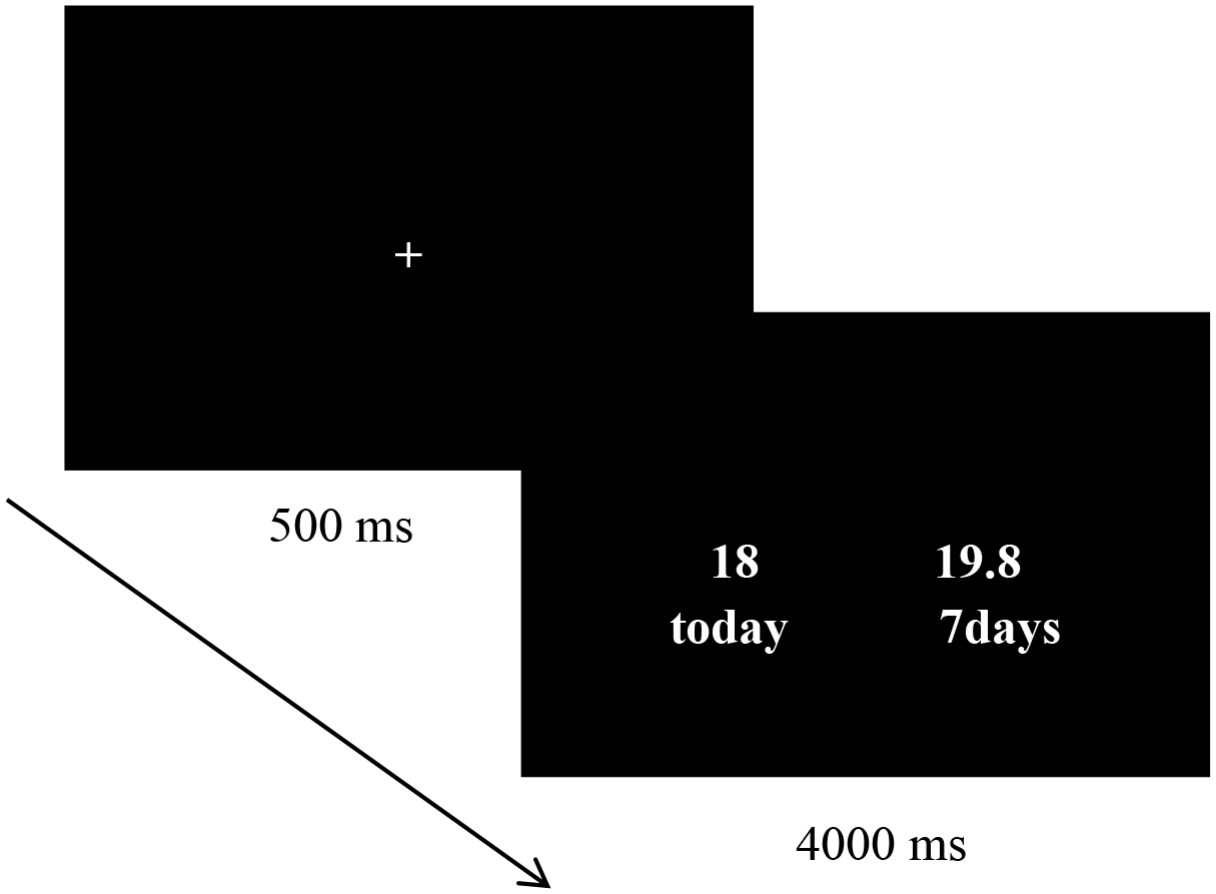
Process diagram of the intertemporal choice task.

In each trial, the fixation was shown at the center of screen for 500 ms, and immediate rewards and delayed rewards were then presented on the left and right sides of screen at the same time for 4000 ms (Fig 2). Participants were asked to choose between the immediate rewards and the delayed rewards. If they preferred the immediate rewards, they were told to press the "F" button. If they preferred the delayed rewards, they were told to press the "G" button. If participants did not press the buttons to make a choice within 4000 ms, the experiment advanced to the next trial automatically.

### Retrospective questions

The Stroop task was immediately followed by three questions asking participants to report the degree of fatigue, effort and depletion that they felt during the Stroop task on a 7-point scale (1 = not at all, 7 = very much) [34] to ensure that the depletion task effectively manipulated the degree of depletion.

1 Do you feel tired after finishing the experiment just now?
1 —— 2 —— 3 —— 4 —— 5 —— 6 —— 7
2 How much effort did you put into resisting the interference of the word meaning?
1 —— 2 —— 3 —— 4 —— 5 —— 6 —— 7
3 Do you feel that your energy is depleted after completing the experiment?
1 —— 2 —— 3 —— 4 —— 5 —— 6 —— 7

After these three questions, participants were asked to report the degree of stress [46] they felt in the Stroop task on a 7-point scale (1 = not at all, 7 = very much), aiming to evaluate the influence of stress on subsequent decision-making behaviors.

Did you feel any pressure during the experiment?
1 —— 2 —— 3 —— 4 —— 5 —— 6 —— 7

Then, participants were asked to complete the Positive and Negative Affect Schedule (PANAS) to assess their mood arousal from the depletion task, which sought to evaluate the influence of emotion on subsequent decision-making behaviors [34]. PANAS is a 5-point scale, including five positive emotional vocabulary words and five negative emotional vocabulary words. The scores for the positive and negative emotional vocabularies were calculated separately, with a higher score representing stronger emotional arousal. In the present study, the Cronbach α coefficient of PANAS was 0.81.

### Equipment

The BIOPAC MP150 (PCO Technology Co., Ltd., Beijing, China) was used to record heartbeat data. The ECG nodes were placed on the left leg, the right leg and the right upper extremity; the left leg was connected to VIN+, the right leg to GND and the right upper extremity VIN-. The ECG channel parameter settings were as follows: sampling rate 2000 Hz; gain 1000 Hz; R-wave NORM; 35 HzLPN ON; HP 0.05 Hz. HRV was measured by calculating the mean squared successive differences in the interbeat interval (RMSSD).

### Procedures

A 2 (trait self-control: high trait self-control, low trait self-control) × 2 (state self-control: high depletion, low depletion) between-subject designed experiment was conducted. The percentage of the selection of immediate rewards (SS%) in the intertemporal decision tasks was recorded as a behavioral indicator, and the participants’ HRV data were recorded as physiological indicators during the resting stage, Stroop task, and intertemporal decision task.

The experiments were performed individually at the physiological polygraph laboratory. The resting HRV was first recorded for 3 minutes. Participants were then asked to complete the Stroop task, followed by three questions to verify the effects of the depletion task, one question evaluating the degree of stress, and PANAS for assessing mood arousal following the depletion task, which required approximately 50 s totally. Immediately thereafter, participants were asked to complete the intertemporal decision task. In this study, participant’s right upper extremity was attached to the node, so all participants were asked to respond with their left hands. The preliminary test and participants’ subjective reports showed that they all completed the experimental tasks smoothly with their left hands. Furthermore, participants were asked to minimize physical movements throughout the experiments, especially in the feet and right hand, while using their left hand to press the button.

## Results

### The depletion effect and control of irrelevant variables

#### Ego depletion effect

The descriptive statistics of the degrees of fatigue, effort and depletion are shown in Table 3.

**Table 3:** Descriptive statistics of the degrees of fatigue, effort and depletion.

	High trait self-control (*M* ± *SD*)		Low trait self-control (*M* ± *SD*)	
	High depletion	Low depletion	High depletion	Low depletion
**Fatigue**	2.14 ± 1.19	2.08 ± 1.24	2.57 ± 1.26	2.62 ± 1.43
**Effort**	4.61 ± 1.37	4.74 ± 1.73	4.50 ± 1.47	4.67 ± 1.63
**Depletion**	2.77 ± 1.16	2.54 ± 1.30	3.10 ± 1.32	2.97 ± 1.29

**Fig 3:**
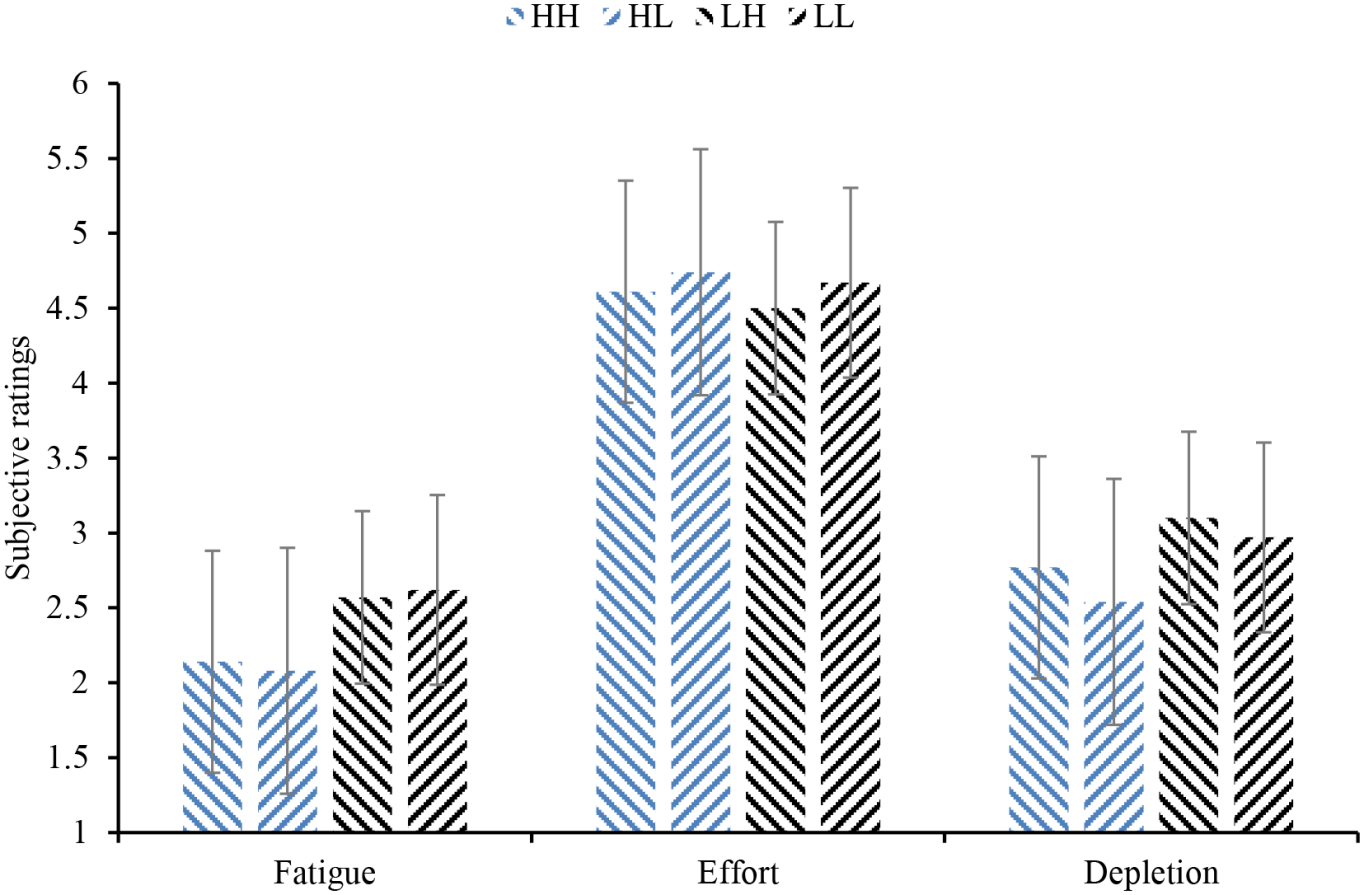
Results of subjective ratings. “HH” represents participants with high trait self-control who finished the high-depletion task; “HL” represents participants with high trait self-control who finished the low-depletion task; “LH” represents participants with low trait self-control who finished the high-depletion task, and “LL” represents participants with low trait self-control who finished the low-depletion task; the same abbreviations are used below and each bar represents the standard error (SE).

For the results regarding the degrees of fatigue, effort and depletion, 2 (trait self-control: high trait self-control, low trait self-control) × 2 (state self-control: high depletion, low depletion) analysis of variance (ANOVA) was conducted (Fig 3). Homogeneity of variance tests were also performed for the degrees of fatigue, effort and depletion (fatigue: *p* = 0.077, effort: *p* = 0.452, depletion: *p* = 0.854), and the results showed that the variances were homogeneous. ANOVA showed that the main effect of trait self-control on the degree of fatigue was significant (*F*(1, 158) = 5.88, *p* = 0.016, η^2^ = 0.04), with participants with low trait self-control (*M* = 2.59, *SD* = 1.34) experiencing a higher level of fatigue than those with high trait self-control (*M* = 2.11, *SD* = 1.21). The main effect of trait self-control on the degree of depletion was marginally significant (*F*(1, 158) = 3.69, *p* = 0.057, η^2^ = 0.02, with low trait self-control participants (*M* = 3.04, *SD* = 1.30) feeling more depleted than high trait self-control participants (*M* = 2.66, *SD* = 1.22). The main effect of trait self-control on the degree of effort (*F*(1, 158) = 0.15, *p* = 0.696) was not significant. The main effects of state self-control on the degree of fatigue (*F*(1, 158) = 0.002, *p* = 0.962), effort (*F*(1, 158) = 0.37, *p* = 0.543) and depletion (*F*(1, 158) = 0.82, *p* = 0.366) were not significant. The interaction effects between trait self-control and state self-control on the degree of fatigue (*F*(1, 158) = 0.06, *p* = 0.805), effort (*F*(1, 158) = 0.01, *p* = 0.940) and depletion (*F*(1, 158) = 0.08, *p* = 0.785) were not significant.

#### Stress

The descriptive statistics of the degree of stress are shown in Table 4.

**Table 4:** Descriptive statistics of the degree of stress.

	High trait self-control (*M* ± *SD*)		Low trait self-control (*M* ± *SD*)	
	High depletion	Low depletion	High depletion	Low depletion
**Stress**	3.00 ± 1.22	2.92 ± 1.69	3.63 ± 1.61	3.03 ± 1.44

To analyze the degree of stress data, 2 (trait self-control: high trait self-control, low trait self-control) × 2 (state self-control: high depletion, low depletion) ANOVA was conducted. Tests for homogeneity variance were performed for the degree of stress, and the results showed that the variance was homogeneous (*p* = 0.245). ANOVA showed that the main effect of trait self-control (*F*(1, 158) = 2.39, *p* = 0.124), the main effect of state self-control (*F*(1, 158) = 2.07, *p* = 0.153), and the interaction effect between trait self-control and state self-control (*F*(1, 158) = 1.23, *p* = 0.268) were not significant for the degree of stress. The results showed that the difficulty of the Stroop task did not affect the degree of stress for individuals with high and low trait self-control.

#### Mood

The descriptive statistics of the emotional experience scores are shown in Table 5.

**Table 5:** Descriptive statistics of scores in positive and negative emotional dimensions.

	High trait self-control (*M* ± *SD*)		Low trait self-control (*M* ± *SD*)	
	High depletion	Low depletion	High depletion	Low depletion
**Positive emotion**	2.63 ± 0.79	2.49 ± 0.93	2.50 ± 0.70	2.44 ± 0.56
**Negative emotion**	1.59 ± 0.60	1.41 ± 0.54	1.57 ± 0.56	1.68 ± 0.56

PANAS consists of the two dimensions of positive emotion and negative emotion, and 2 (trait self-control: high trait self-control, low trait self-control) × 2 (state self-control: high depletion, low depletion) multivariate ANOVA was conducted on the positive and negative emotional scores. First, Box’s test was performed for the positive and negative emotional scores, and the results showed that the covariance matrix was homogeneous (*p* = 0.314). The results of multivariate ANOVA indicated that the main effect of trait self-control (*F*(1, 158) = 0.56, *p* = 0.455), the main effect of state self-control (*F*(1, 158) = 0.66, *p* = 0.417), and the interaction effect between trait self-control and state self-control (*F*(1, 158) = 0.10, *p* = 0.750) were not significant for positive emotional experiences. The main effect of trait self-control (*F*(1, 158) = 1.92, *p* = 0.168), the main effect of state self-control (*F*(1, 158) = 0.17, *p* = 0.679), and the interaction effect between trait self-control and state self-control (*F*(1, 158) = 2.62, *p* = 0.107) were not significant for negative emotional experiences. The results showed that the difficulty of the Stroop task did not affect the emotion of individuals with high and low trait self-control.

#### Additional variables

The independent sample *t* test was used to examine the gender effect on SS% and reaction time during the intertemporal decision task, and the results showed that there were no significant differences in SS% (*t*(160) = 0.09, *p* = 0.927) and reaction time (*t*(160) = 0.92, *p* = 0.361) between males and females. In addition, the correlations of mood and SS%; mood and reaction time; stress and SS%; and stress and reaction time were analyzed, and the results showed that there were no significant correlations between positive emotional experiences and SS% (*r* = 0.15, *p* = 0.063); positive emotional experiences and reaction time (*r* = 0.02, *p* = 0.765); negative emotional experiences and SS% (*r* = 0.12, *p* = 0.115); negative emotional experiences and reaction time (*r* = –0.001, *p* = 0.991); stress and SS% (*r* = –0.04, *p* = 0.601); and stress and reaction time (*r* = 0.04, *p* = 0.651).

### Behavioral data analysis and results

#### Stroop task

The descriptive statistics of accuracy (ACC) and response time (RT) during the Stroop task are shown in Table 6.

**Table 6:** Descriptive statistics of accuracy and response time during the Stroop task.

	High trait self-control (*M* ± *SD*)		Low trait self-control (*M* ± *SD*)	
	High depletion	Low depletion	High depletion	Low depletion
**ACC (%)**	96.80 ± 3.37	98.38 ± 1.60	96.87 ± 3.63	98.36 ± 2.06
**RT (ms)**	805.63 ± 139.66	730.46 ± 90.61	798.40 ± 96.53	705.59 ± 95.04

For the results regarding accuracy and reaction time in the Stroop task, 2 (trait self-control: high trait self-control, low trait self-control) × 2 (state self-control: high depletion, low depletion) ANOVA was conducted. Homogeneity tests of variance were performed for accuracy and response time (accuracy: *p* = 0.005, reaction time: *p* = 0.076). The results showed that the variance of reaction time was homogeneous, but the variance of accuracy was not homogeneous. Therefore, a post hoc test of heterogeneous variance was conducted for accuracy. ANOVA indicated that during the Stroop task, the main effect of state self-control on accuracy was significant (*F*(1, 158) = 11.96, *p* = 0.001, η^2^ = 0.07). Participants’ accuracy was higher when completing low-depletion tasks (*M* = 98.37, *SD* = 1.83) than when completing high-depletion tasks (*M* = 96.83, *SD* = 3.47) (*t*(127.774) = –3.56, *p* = 0.001, *d* = 0.55). Neither the main effect of trait self-control (*F*(1, 158) = 0.004, *p* = 0.952) nor the interaction effect between trait self-control and state self-control (*F*(1, 158) = 0.01, *p* = 0.906) on accuracy was significant. The main effect of state self-control on reaction time was significant (*F*(1, 158) = 24.25, *p* < 0.001, η^2^ = 0.13). Participants responded faster in low-depletion tasks (*M* = 718.02, *SD* = 93.09) than in high-depletion tasks (*M* = 802.19, *SD* = 120.40). The main effect of trait self-control on reaction time was not significant (*F*(1, 158) = 0.89, *p* = 0.348), nor was the interaction effect between trait self-control and state self-control on reaction time (*F*(1, 158) = 0.27, *p* = 0.606).

### Intertemporal decision tasks

The descriptive statistics of SS% and RT during the intertemporal decision task are shown in Table 7.

**Table 7:** Descriptive statistics of SS% and RT during the intertemporal choice task.

	High trait self-control (*M* ± *SD*)		Low trait self-control (*M* ± *SD*)	
	High depletion	Low depletion	High depletion	Low depletion
**SS%**	33.06 ± 17.71	32.15 ± 18.91	50.71 ± 21.72	36.08 ± 18.64
**RT**	1593.46 ± 409.10	1376.14 ± 367.25	1397.40 ± 451.82	1387.39 ± 471.38

**Fig 4:**
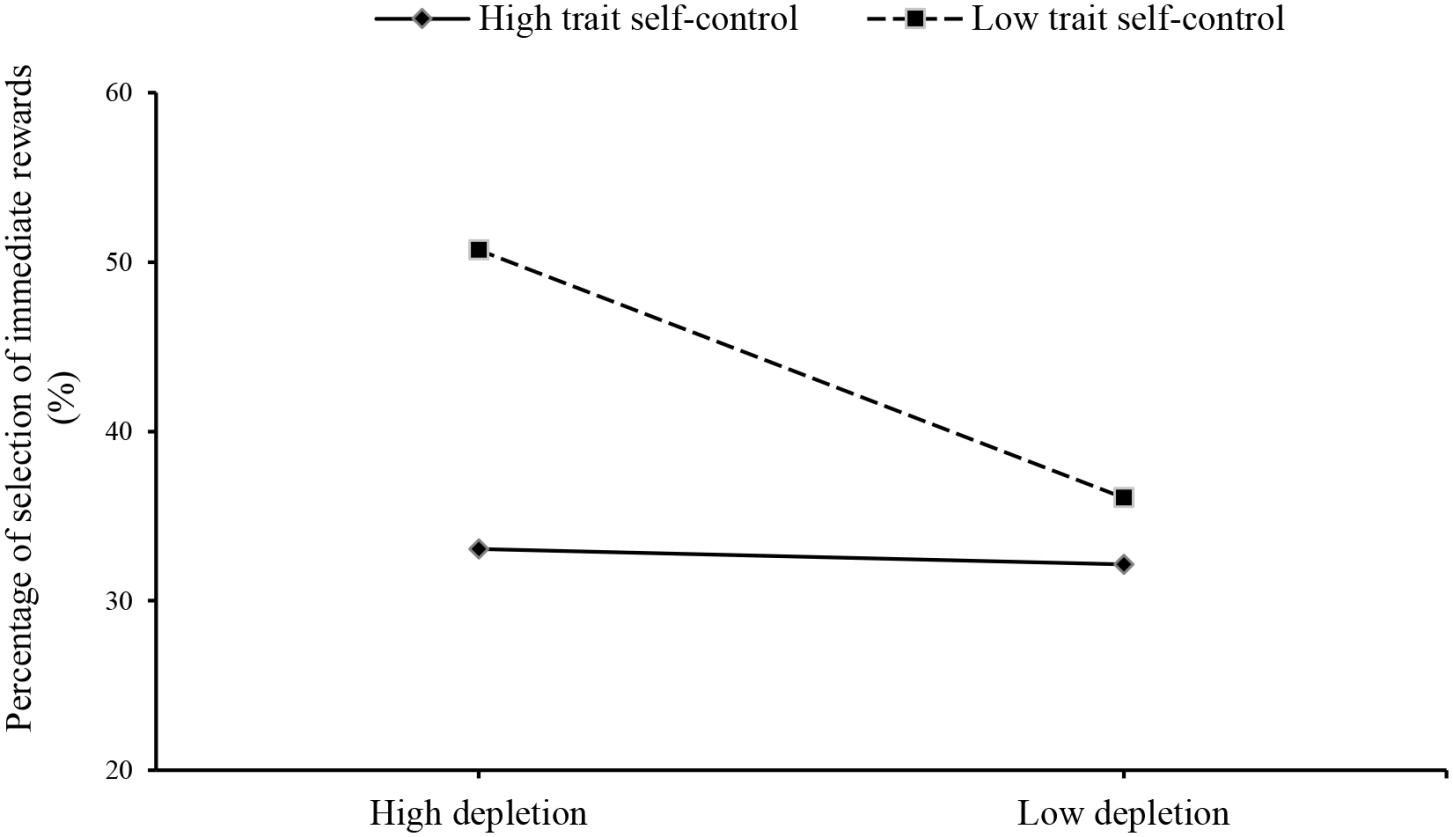
Percentage of selection of immediate rewards (%).

For the results regarding SS% and RT in intertemporal decision tasks, 2 (trait self-control: high trait self-control, low trait self-control) × 2 (state self-control: high depletion, low depletion) ANOVA was conducted (Fig 4). Homogeneity of variance tests were performed for SS% and response time in the four groups (SS%: *p* = 0.295, reaction time: *p* = 0.603), and the results showed that the variances were homogeneous. ANOVA indicated that the main effect of trait self-control on SS% was significant (*F*(1, 158) = 12.67, *p* < 0.001, η^2^ = 0.07), with participants with low trait self-control (*M* = 43.49, *SD* = 21.43) being more likely to choose immediate rewards than participants with high trait self-control (*M* = 32.63, *SD* = 18.18). The main effect of state self-control on SS% was significant (*F*(1, 158) = 6.57, *p* = 0.011, η^2^ = 0.04), with participants showing a greater preference for immediate rewards after completing high-depletion tasks (*M* = 41.46, *SD* = 21.51) than after low-depletion tasks (*M* = 34.11, *SD* = 18.76). The interaction effect between trait self-control and state self-control on SS% was significant (*F*(1, 158) = 5.12, *p* = 0.025, η^2^ = 0.03), and simple-effect analysis showed that the percentage of the selection of immediate rewards among participants with low trait self-control was higher after completing high-depletion tasks than after low-depletion tasks (*F*(1, 158) = 10.81, *p* = 0.001, η^2^ = 0.07). The main effect of trait self-control (*F*(1, 158) = 1.90, *p* = 0.170), the main effect of state self-control (*F*(1, 158) = 2.87, *p* = 0.092) and the interaction effect between trait self-control and state self-control (*F*(1, 158) = 2.39, *p* = 0.124) on reaction time were all nonsignificant.

### Physiological data analysis and results

Offline data analysis of HRV was carried out using Acknowledge software, and SPSS 20.0 was employed for further statistical analysis. The descriptive statistics of HRV in the stages of resting, depletion and decision making are shown in Table 8.

**Table 8:** Descriptive statistics of HRV in the stages of resting, depletion and decision making.

	High trait self-control (*M* ± *SD*)		Low trait self-control (*M* ± *SD*)	
	High depletion	Low depletion	High depletion	Low depletion
**Resting**	42.25 ± 18.53	38.61 ± 15.73	35.49 ± 14.01	34.25 ± 12.49
**Depletion**	41.23 ± 20.96	36.96 ± 18.11	34.51 ± 14.00	33.13 ± 10.89
**Decision making**	40.07 ± 21.34	36.14 ± 18.30	32.96 ± 12.33	32.79 ± 10.60

**Fig 5:**
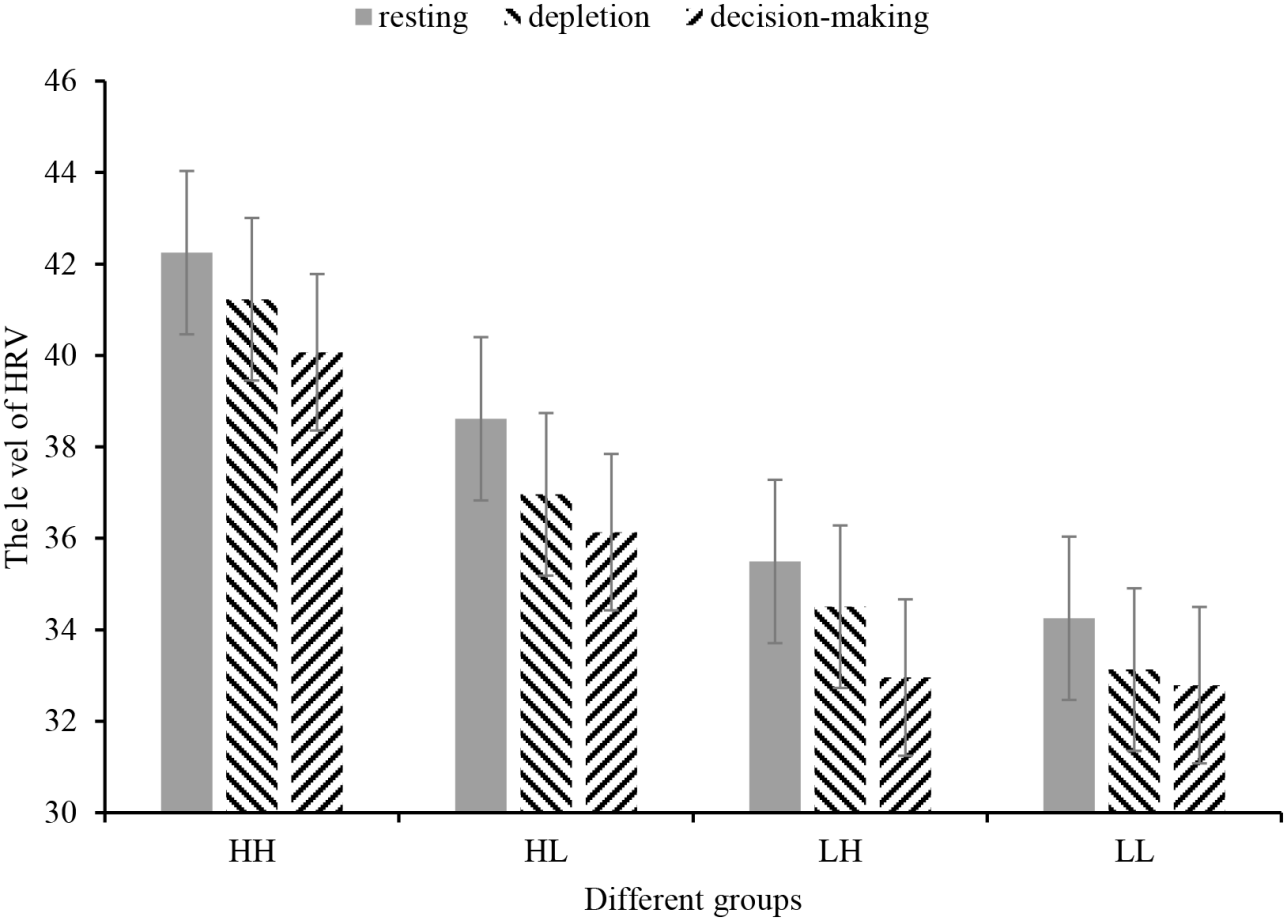
HRV among different groups in different experimental stages.

For the results regarding HRV, 2 (trait self-control: high trait self-control, low trait self-control) × 2 (state self-control: high depletion, low depletion) × 3 (experimental stage: resting, depletion, intertemporal decisions) mixed-design ANOVA was conducted, and we treated gender as a covariate to dismiss the alternative explanation that the effect stems from imbalanced gender ratios among the four groups (Fig 5). The Mauchly spherical test was carried out, and the results showed that HRV did not obey the spherical hypothesis. Therefore, Greenhouse-Geisser correction was adopted to correct the degree of freedom. ANOVA indicated that the main effect of experimental stage on HRV was not significant (*F*(1.86, 292.54) = 0.04, *p* = 0.95). The main effect of trait self-control on HRV was significant (*F*(1, 157) = 6.51, *p* = 0.012, η^2^ = 0.04), high trait self-control participants exhibiting (*M* = 39.62, *SD* = 1.69) a significantly higher HRV than participants with low trait self-control (*M* = 33.43, *SD* = 1.73) throughout the experimental stages. The main effect of state self-control (*F*(1, 157) = 1.24, *p* = 0.268) and the interaction effects between experimental stage and trait self-control (*F*(1.86, 292.54) = 0.09, *p* = 0.913), experimental stage and state self-control (*F*(1.86, 292.54) = 0.20, *p* = 0.816), and trait self-control and state self-control (*F*(1, 157) = 0.19, *p* = 0.667) as well as the interaction effects among experimental stage, trait self-control and state self-control (*F*(1.86, 292.54) = 0.21, *p* = 0.800) were all nonsignificant.

## Discussion

Stroop tasks with different levels of difficulty were employed as depletion tasks to manipulate the state of self-control. To examine the validity of Stroop tasks as depletion tasks, participants were asked to report the extent of fatigue, effort, and depletion that they experienced during the Stroop task. The results showed that although participants with high and low trait self-control made the same effort to complete the Stroop task, participants with low trait self-control felt more tired and more depleted during the Stroop task than participants with high trait self-control. Furthermore, participants with high and low trait self-control exhibited the same tendencies regarding accuracy and response time in the Stroop task. The accuracy of both groups was lower in high-depletion tasks than in low-depletion tasks, and their reaction was slower in high-depletion tasks than in low-depletion tasks. To some extent, these results verified that the difficulty of the depletion tasks was manipulated effectively. Previous studies have shown that the Stroop task can also be used to manipulate the degree of stress [46], and the emotional state of participants might affect intertemporal decisions [47]. Participants were asked to report the degree of stress and their emotional state after the Stroop task and to assess the interference of these factors in their intertemporal decisions. Data analysis showed that the degree of stress and emotion that participants experienced in the Stroop task did not significantly affect their performance in the intertemporal decision task.

The behavioral results for intertemporal decision tasks showed that the percentage of the selection of immediate rewards was significantly lower among participants with high trait self-control than among participants with low trait self-control. Low trait self-control participants’ decisions were affected by the difficulty of the depletion tasks, and they were more likely to choose immediate rewards after the high-depletion task than after the low-depletion task. However, the preference for immediate rewards was not affected by the depletion task among the high trait self-control participants. Previous research has shown that compared with individuals with high trait self-control, individuals with low trait self-control are more prone to seek out information and situations that might easily trigger their impulsivity [14, 39], and taking attention away from impulsive cues requires consumption of self-regulatory resources [40]. Therefore, compared with the low-depletion task, the high-depletion task consumed more self-regulatory resources among participants with low trait self-control, and their remaining self-regulatory resources were not sufficient to distract their attention from impulsive cues, such as the immediate payment time. These particular mental processes involved in making intertemporal decisions among participants with low trait self-control might explain the vulnerability of their self-control capability after accomplishing depletion tasks. Therefore, these participants preferred more immediate rewards after high-depletion tasks than low-depletion tasks.

Trait self-control is considered to be a stable personality trait [19]. Trait HRV reflects the inhibitory ability of the CAN [24–25], in which the prefrontal cortex exerts an inhibitory effect on subcortical brain structures [20]. Individuals with a certain level of trait self-control maintain a relatively stable inhibitory capacity, and individuals with high trait self-control are usually better at controlling impulsive behavior than individuals with low trait self-control [14, 18]. Therefore, throughout experimental stages, the participants with high trait self-control exhibited a significantly higher HRV and more stable preferences for immediate rewards than participants with low trait self-control. However, the interaction effects among experimental stages, trait and state on HRV were not significant. There were no significant differences in HRV during tasks between the participants with low trait self-control who completed high-depletion tasks and those with low trait self-control who completed low-depletion tasks. Additionally, no significant differences in HRV were found during experimental tasks between the participants with high trait self-control who completed high-depletion tasks and those with high trait self-control who performed low-depletion task, in the stages of the depletion tasks and intertemporal decision tasks. These non-significant results were consistent with the participants’ reports of the extent of fatigue, effort, and depletion they experienced in the Stroop task. The participants with low trait self-control who completed high-depletion tasks reported that they felt the same degrees of fatigue, effort, and depletion as those with low trait self-control who performed low-depletion tasks, which was also observed for the participants with high trait self-control who carried out both high- and low-depletion tasks. These results are consistent with some previous studies [48–50] on the relationship between the transient variation of HRV during tasks and the variation of the amount of self-regulatory resources during experimental tasks [24]. It has been found that there may be a positive correlation [51–54], or negative correlation [55–56], or even no correlation [48–50] as shown by the results of the present research. These mixed relationships might have been caused by different types of experimental tasks conducted to manipulate self-regulatory resources [24]. Tasks involving emotional regulation [53–54], execution function, and working memory [51–52] have been shown to activate different mental processes in previous studies. Therefore, we posit that a possible reason for the non-significant interaction effects among experimental stages, trait and state on HRV is that the Stroop task and the intertemporal decision task both involve more than one mental processing mechanism. The accomplishment of Stroop tasks requires the cognitive process to perceive the painting color and self-control to suppress the dominant response to the meaning of the word. Self-control, cognitive and emotional processes are all activated when an individual makes intertemporal decisions. The prefrontal cortex is a common neural basis of self-control and cognitive processes. When the prefrontal cortex is deactivated, executive function in subsequent cognitive processing and self-control tasks is damaged [57]. For example, individuals prefer more immediate gains after the N-back working memory task, leading to transient suppression of activity in the prefrontal cortex [58]. An intertemporal decision requires cognitive resources to calculate the subjective probability that the delayed rewards will be delivered as promised [57]. An emotional process is also activated in intertemporal decisions. Delayed rewards are regarded as unwelcome and unexpected results [59], and the amplitude of feedback-related negativity caused by delayed rewards is greater than that caused by immediate rewards [60]. In addition to the self-control component, an intertemporal decision involves cognitive and emotional components. The correlations may not be consistent between the transient variations of HRV during tasks and the multiple mental and neural processes activated by the Stroop task and the intertemporal decision task in the present study, which could lead to failure in the identification of significant differences in HRV during tasks between the two groups of participants with certain levels of trait self-control who completed high-depletion and low-depletion tasks.

Moreover, these results strongly imply that focusing on the mental mechanisms activated in making intertemporal decisions in future research may be a better way to further clarify the mental mechanism of how state self-control influences the intertemporal decisions made by individuals with high and low trait self-control. Previous studies have shown that different cognitive processes are adopted by high temporal discounters *versus* low temporal discounters in completing an intertemporal decision task. Compared with low temporal discounters, high temporal discounters produce greater reward positivity for immediate rewards and overvalue immediate rewards [14]. Schmidt et al. [16] found that individuals with low trait self-control produce a greater amplitude of reward positivity for the differences between immediate and delayed rewards than individuals with high trait self-control. Studies on neural mechanisms have shown that enhanced structural integrity of white matter fiber bundles between prefrontal and striatal brain areas is associated with better impulse control [61]. When making intertemporal decisions, the structural and functional connectivity between the striatum and the lateral prefrontal cortex is associated with increased patience, whereas connectivity between subcortical areas and the striatum is associated with increased impulsivity [62]. High discounters show lower neural activity in the ventromedial prefrontal cortex (VMPFC) and the ventral striatum (VS) for longer time delays, while low discounters show the opposite pattern [63]. Therefore, participants with high and low trait self-control may employ different mental and neural processing mechanisms to accomplish intertemporal decision tasks. Further studies may need to focus on psychological and neural mechanisms to explore the state of self-control in intertemporal decisions between individuals with high and low trait self-control.

## Conclusions

To determine how state self-control influences the intertemporal decisions made by individuals with high and low trait self-control, the Stroop task was used as a depletion task to manipulate the state of self-control. It was determined that participants with high trait self-control are more likely to choose delayed rewards and exhibit a higher HRV than participants with low self-control. Participants with low trait self-control are more willing to choose immediate rewards, and their preferences for immediate rewards are more vulnerable to the difficulty of depletion tasks. These findings suggest that the participants with high trait self-control exhibit greater and more stable self-control abilities than participants with low trait self-control.
